# The Value of Combining Serum Alanine Aminotransferase Levels and Body Mass Index to Predict Mortality and Medical Costs: a 10-year Follow-up Study of National Health Insurance in Shiga, Japan

**DOI:** 10.2188/jea.16.15

**Published:** 2005-12-20

**Authors:** Koshi Nakamura, Tomonori Okamura, Hideyuki Kanda, Takehito Hayakawa, Akira Okayama, Hirotsugu Ueshima

**Affiliations:** 1 Department of Health Science, Shiga University of Medical Science.; 2 Department of Hygiene and Preventive Medicine, Fukushima Medical University.; 3 Department of Public Health Science, Shimane University School of Medicine.; 4 Department of Preventive Cardiology, National Cardiovascular Center.

**Keywords:** Alanine Transaminase, Body Mass Index, Mortality, Health Expenditure

## Abstract

**BACKGROUND:**

Evidence suggests that the predictive value of serum alanine aminotransferase (ALT) levels for prognosis, measured by indices such as all-cause mortality and medical costs, may be modified by body mass index (BMI). However, the relationship between serum ALT and BMI has not been satisfactorily elucidated.

**METHODS:**

Four thousand, five hundred and twenty-four community dwelling Japanese National Health Insurance beneficiaries, 40-69 years old, were classified into five categories according to their serum ALT levels (IU/L) (ALT<20, 20≤ALT<30, 30≤ALT<40, 40≤ALT<50 and 50≤ALT) and followed for 10 years. Hazard ratios for all-cause mortality, with reference to the lowest serum ALT category, and medical costs per person were evaluated for each serum ALT category after analyzing interactions between serum ALT levels and BMI for all-cause mortality and for medical costs.

**RESULTS:**

A significant interaction between serum ALT levels and BMI was observed. In participants below the median BMI, positive, graded relationships were identified between serum ALT levels and all-cause mortality as well as between serum ALT levels and personal medical costs. The multivariate-adjusted hazard ratio in the “50≤ALT” category showed an approximately 8-fold increase. However, in the participants at or above the median BMI, no significant relationships between serum ALT levels and all-cause mortality or personal medical costs were identified.

**CONCLUSIONS:**

In these Japanese participants, the predictive value of serum ALT levels for prognosis was more evident if BMI was taken into account. A combination of high serum ALT levels and below median BMI was associated with excess mortality and medical costs.

Serum alanine aminotransferase (ALT) levels are useful indicators for certain liver diseases, such as viral hepatitis, alcoholic hepatitis, and fatty liver.^1^ The serum ALT level is positively associated with all-cause or liver disease mortality,^2^ even for ALT values within the clinically normal range.^3^ Fatty liver is the most common liver disease and is associated with a slight-to-moderate elevation in serum ALT levels,^1^ especially in individuals with a high body mass index (BMI).^4^ Fatty liver alone rarely leads to an increase in mortality, because hepatic failure typically occurs only in a few such patients.^1^^,^^5^ On the other hand, recent studies have shown that fatty liver, which is a marker of visceral fat, is associated with the metabolic syndrome,^6^^,^^7^ a major risk factor for cardiovascular disease.^8^ Therefore, the prognostic value of serum ALT levels might be modified by BMI.

The objectives of the present study were as follows: (1) to clarify the predictive value of serum ALT level for all-cause mortality and for medical costs (a possible marker of chronic, non-fatal liver disease); and (2) to examine whether BMI modified the predictive value of the serum ALT level. To our knowledge, no such cohort study has ever been conducted.

## METHODS

### Study Design and Participants

A cohort study of Japanese National Health Insurance (NHI) beneficiaries residing in a single geographic area was undertaken with the objective of clarifying the relationships between baseline serum ALT levels and all-cause mortality or medical costs. Medical insurance is compulsory for everyone living in Japan and comprises two systems: the first system is for employees and their dependants; while the second system, the NHI, is for self-employed people (such as farmers and fishermen) and retirees, along with their dependants.^9^ The NHI covers 34.7% of the overall Japanese population.^9^

The participants in the study cohort were 4,524 NHI beneficiaries, 40-69 years old, living in seven rural towns and one village in Shiga Prefecture, West Japan, who underwent a baseline survey in 1989-1991. In 1990, the study area had 82,155 residents, including 31,564 individuals aged 40-69 years old, of whom 11,900 were NHI beneficiaries.^10^ Therefore, the study participants represented approximately 38% of all NHI beneficiaries aged 40-69 years old in the area. The present study was performed as part of the research work of the Health Promotion Research Committee of the Shiga NHI Organizations. NHI claims were linked with the baseline survey data files at the Shiga NHI Organizations. The participants’ names were deleted from the linked data at the Shiga NHI Organizations, thereby protecting the confidentiality of the participants’ identities during data analysis. The present study was approved by the Institutional Review Board of Shiga University of Medical Science for ethical issues (No. 16-15).

### Data Collection

The baseline survey in 1989-1991 was performed using standardized methods according to the Manual for Health Check-ups under the Medical Service Law for the Aged issued by the Japan Public Health Association in 1987.^11^ Serum ALT levels were measured by an ultraviolet method. Body height and body weight were measured, and BMI was calculated as body weight (kg) divided by the square of body height (m). Smoking and drinking habits, medications for hypertension, and diabetes mellitus history were evaluated from interviews with well-trained public health nurses. Ex-drinkers were classified as non-drinkers in the present study because we had no information on past drinking history. Blood pressure was measured once using a standard mercury sphygmomanometer on the right arm of each participant in the sitting position after at least a 5-minute rest. Serum total cholesterol levels were measured by an enzymatic method.

The participants were classified into five categories according to their baseline serum ALT levels (IU/L): “ALT<20”, “20≤ALT<30”, “30≤ALT<40”, “40≤ALT<50”, and “50≤ALT”. In each serum ALT category, we evaluated the hazard ratio for all-cause mortality and medical costs per person after a 10-year follow-up.

We obtained information on beneficiaries who died or those who withdrew from the NHI system and the medical insurance costs for each participant from April in the year following the health check-ups until March 2001 using the monthly NHI claim history files of the Shiga NHI Organizations. Costs were expressed in Japanese *Yen* (i.e. 100 Japanese *Yen* = 0.91 US Dollars or 0.73 Euro, at the foreign exchange rate on September 1, 2005). Medical costs data for each participant differed depending upon the period of subscription to the NHI. Therefore, the medical cost for each participant was divided by the period of subscription, and expressed as costs per month of follow-up. If a beneficiary died or withdrew, follow-up was stopped at that point; if a beneficiary who had withdrawn subsequently re-enrolled in the NHI, follow-up was re-started. Reasons for withdrawal from the NHI included moving to areas outside of Shiga Prefecture and/or transfer to another insurance system.

### Data Analysis

A Cox proportional hazards model was used to examine the association between serum ALT category or log-transformed serum ALT levels (test for linear trend) and all-cause mortality. The hazard ratio for all-cause mortality in each serum ALT category was compared to the lowest serum ALT category (“ALT<20”). This model incorporated the following variables as covariates: age, sex, BMI, smoking habit (non-smoker or current smoker), drinking habit (non-drinker, current occasional drinker, or current daily drinker, using two dummy variables and “non-drinker” as a reference), systolic blood pressure, medication for hypertension, serum total cholesterol and history of diabetes mellitus.

Personal total medical costs for each serum ALT category, separated into outpatient and inpatient costs, were expressed using arithmetic means. Furthermore, the cost data were logarithmically transformed to normalize the distribution, with the results expressed as geometric means, because the distribution of real medical costs revealed positive skewness. For the participants with 0 *Yen* (per month), the logarithmic transformations were performed by replacing 0 *Yen* with 1 *Yen*. There were 15 participants with total medical costs of 0 *Yen* and 16 participants with outpatient medical costs of 0 *Yen*. For comparisons of total and outpatient medical costs per person in each serum ALT category, we performed an analysis of covariance, using the same covariates listed above. Because there were 2,600 participants (57.5%) with inpatient medical costs of 0 *Yen*, logarithmic transformations were not performed, and the Kruskal-Wallis test was used to compare inpatient medical costs in each serum ALT category.

The significance of an interaction between serum ALT levels and BMI was tested for all-cause mortality and for total medical costs using an interaction term for the categorical variables in each multivariate-adjusted model. Evaluation of the relationships between serum ALT levels and all-cause mortality or medical costs was carried out for each subgroup, stratified by median BMI if a significant interaction between serum ALT levels and BMI was observed.

If many participants in some subgroups of serum ALT or BMI had a sub-clinical disease not to be detected by the baseline survey, stratifying the follow-up period may change the mortality trends identified by data analysis. The analyses were repeated after dividing the entire follow-up period into a first half and a latter half.

The statistical analysis package SPSS^®^ 11.0J for Windows was used for statistical processing. All probability values were two-tailed, and the significance level was set at P<0.05.

## RESULTS

The baseline risk characteristics of the 4,524 participants, grouped according to their serum ALT levels, are summarized in Table 1. The prevalence of clinically high levels of serum ALT (40≤ALT) was 2.1%. We observed significant differences between serum ALT categories for most of the risk factors. The mean levels of BMI and blood pressure were lowest in the “ALT<20” category; whereas the mean level of serum total cholesterol was lowest in the “50≤ALT” category.

**Table 1.** 1989 -1991 baseline risk characteristics of 4,524 National Health Insurance participants, in Shiga, Japan, grouped by serum alanine aminotransferase.

	Serum ALT (IU/L) category					P value

	ALT<20	20≤ALT<30	30≤ALT<40	40≤ALT<50	50≤ALT	
Number of participants	3,888	439	100	50	47	
(Distribution (%))	(86.0)	(9.7)	(2.2)	(1.1)	(1.0)	
Age (yr)*	54.3 ± 8.1	54.6 ± 8.0	52.9 ± 8.7	51.4 ± 8.4	53.9 ± 8.3	0.047
Sex^†^
Men (%)	39.0	65.6	65.0	62.0	66.0	<0.01
Women (%)	61.0	34.4	35.0	38.0	34.0	
Serum ALT (IU/L)*	11.4 ± 3.5	23.5 ± 2.8	33.6 ± 2.7	43.5 ± 2.9	72.7 ± 27.0	<0.01
Body mass index (kg/m^2^)*	22.6 ± 2.7	24.1 ± 3.1	24.7 ± 3.2	24.8 ± 3.7	23.8 ± 3.0	<0.01
Smoking habit^†^
Non-smoker (%)	74.1	60.9	59.0	63.3	64.4	<0.01
Current smoker (%)	25.9	39.1	41.0	36.7	35.6	
Drinking habit^†^
Non-drinker (%)	57.0	36.7	40.2	43.7	45.6	<0.01
Current occasional drinker (%)	18.2	21.6	16.5	14.6	26.1	
Current daily drinker (%)	24.8	41.7	43.3	41.7	28.3	
Systolic blood pressure (mmHg)*	132.0 ± 17.5	136.6 ± 17.6	139.8 ± 20.2	133.3 ± 14.8	135.6 ± 14.1	<0.01
Diastolic blood pressure (mmHg)*	79.7 ± 10.4	83.2 ± 10.8	84.3 ± 11.4	82.6 ± 10.8	82.7 ± 9.6	<0.01
Medication for hypertension (%)^†^	6.8	9.3	17.0	4.0	4.3	<0.01
Serum total cholesterol (mg/dl)*	196.2 ± 34.7	200.4 ± 39.2	206.9 ± 41.1	197.2 ± 33.6	187.6 ± 50.9	<0.01
History of diabetes mellitus (%)^†^	2.9	3.9	5.0	2.0	2.1	0.58

* One way analysis of variance

† Chi square test

Values located after the mark, ±, indicate standard deviation.

Abbreviations: ALT, alanine aminotransferase

Both all-cause mortality and personal medical costs tended to increase in the higher serum ALT categories, in both the age and sex-adjusted models as well as in the multivariate-adjusted model (data not shown). The interaction for all-cause mortality and for total medical costs between serum ALT levels and BMI was significant in each multivariate-adjusted model (P=0.01, both). Because the proportion of participants in each of the serum ALT groups did not significantly vary between male and female participants, we did not stratify our data by sex; we grouped male and female participants together in our analysis of the relationships between serum ALT levels and all-cause mortality or medical costs, stratified by median BMI.

For the participants below median BMI, after adjusting for age and sex, positive, graded relationships were observed between serum ALT levels and all-cause mortality, as well as between serum ALT levels and personal medical costs; the geometric means of total medical costs in the “ALT<20”, “20≤ALT<30”, “30≤ALT<40”, “40≤ALT<50” and “50≤ALT” categories were 7,887 *Yen* per month, 8,333 *Yen*, 14,059 *Yen*, 19,772 *Yen*, and 30,977 *Yen*, respectively (P<0.01). After multivariate adjustment, a similar pattern of positive, graded relationships was still observed between serum ALT levels and all-cause mortality, as well as between serum ALT levels and personal medical costs; the hazard ratio of all-cause mortality in the “50≤ALT” category showed an approximately 8-fold increase which had statistical significance (Table 2).

**Table 2.** Hazard ratios for all-cause mortality and medical costs per person grouped by serum alanine aminotransferase and stratified by median of body mass index, after 10-year follow-up from 1990 to 2001, in National Health Insurance in Shiga, Japan.

Serum ALT (IU/L) category	Number of participants	All-cause mortality		Medical costs per person (Japanese *Yen* per month)				

				Total		Outpatient		Inpatient	
			
		Number	Hazard ratio^‡^	Arithmetic mean	Geometric mean^§^	Arithmetic mean	Geometric mean^§^	Arithmetic mean^¶^
Participants below median BMI (22.7 (kg/m^2^))								
ALT<20	2,056	100	1.00	19,882	7,864	10,340	5,837	9,566
20≤ALT<30	146	14	1.42 [0.80-2.52]	26,752	8,136	12,129	5,597	14,622
30≤ALT<40	28	1	0.91 [0.13-6.55]	46,559	13,227	15,970	9,367	30,956
40≤ALT<50	13	2	3.53 [0.85-14.61]	33,276	20,414	14,987	12,308	18,288
50≤ALT	17	5	8.11 [3.16-20.82]	62,956	29,882	23,542	16,010	39,414
			P for trend<0.01		P<0.01		P<0.01	P<0.01
Participants at or above median BMI (22.7 (kg/m^2^))								
ALT<20	1,832	78	1.00	19,391	8,866	11,196	6,581	8,188
20≤ALT<30	293	6	0.33 [0.14-0.77]	17,926	8,699	11,404	6,857	6,522
30≤ALT<40	72	4	0.88 [0.31-2.49]	20,382	10,883	14,601	8,639	5,781
40≤ALT<50	37	1	0.64 [0.09-4.65]	17,567	11,204	12,825	9,443	4,781
50≤ALT	30	3	1.38 [0.34-5.63]	23,640	9,605	13,264	6,667	10,376
			P for trend=0.91		P=0.49		P=0.10	P=0.32

‡ Multivariate analysis using a Cox proportional hazards regression model, adjusted for age, sex, BMI, smoking habit, drinking habit, systolic blood pressure, medication for hypertension, serum total cholesterol and history of diabetes mellitus.

§ Multivariate analysis of covariance, adjusted for age, sex, BMI, smoking habit, drinking habit, systolic blood pressure, medication for hypertension, serum total cholesterol and history of diabetes mellitus.

¶ Kruskal-Wallis test

Values in brackets indicate 95% confidence interval of hazard ratio.

Abbreviations: ALT, alanine aminotransferase; BMI, body mass index

On the other hand, for the participants at or above the median BMI, after adjusting for age and sex, no significant relationships were observed between serum ALT levels and all-cause mortality or personal medical costs; the geometric means of total medical costs in the “ALT<20”, “20≤ALT<30”, “30≤ALT<40”, “40≤ALT<50”, and “50≤ALT” categories were 8.725 *Yen* (per month), 9,082 *Yen*, 13.108 *Yen*, 11,603 *Yen*, and 9,614 *Yen*, respectively (P=0.06). After multivariate adjustment, significant relationships were still not observed (Table 2). When we performed the analyses after dividing the entire follow-up period into first half and latter half periods, the results for each period had patterns generally similar to those shown in Table 2 (data not shown).

## DISCUSSION

The strengths of the present cohort study are that our study outcomes included not only all-cause mortality, but also medical costs. In addition, we analyzed the interaction between serum ALT levels and BMI with respect to prognosis. We demonstrated that taking BMI into account affected the predictive value of serum ALT levels in a surprising fashion. We observed positive, graded relationships between serum ALT levels and all-cause mortality as well as between serum ALT levels and personal total medical costs for participants with below median BMI, whereas we observed no significant relationships for participants at or above the median BMI.

Visceral fat accumulation is a major component of the metabolic syndrome.^12^ For the Japanese population, proposed cut-off points for the metabolic syndrome have been established at above 85 cm in men and 90 cm in women, which are lower than those for Western populations.^13^ Fatty liver, a consequence of visceral fat accumulation, is also associated with the metabolic syndrome.^6^^,^^7^ Fatty liver is frequently found in obese individuals with high serum ALT levels.^4^ Therefore, we had hypothesized that high levels of both serum ALT and BMI might lead to increases in mortality and medical costs via the metabolic syndrome, although we had no information about our participants regarding waist circumference. However, our study found that high serum ALT levels were not significantly associated with increased overall mortality or increased medical costs in participants at or above the median BMI, which was contrary to the expectations of our disease model. One possible explanation for this discrepancy is that because the mean level of BMI in the Japanese population is much lower than in Western populations,^14^ total mass of visceral fat may still have been relatively small, even in the higher BMI group in our study population. Furthermore, the incidence or mortality of atherosclerotic cardiovascular disease due to the metabolic syndrome, such as coronary heart disease and the large artery occlusive type of cerebral infarction,^15^ is lower in the Japanese population compared with Western populations,^15^^-^^20^ whereas the incidence or mortality of intra-cerebral hemorrhage or the lacunar type of cerebral infarction is higher in the Japanese population.^15^^,^^19^^-^^21^

High serum ALT levels were associated with increases in mortality and medical costs in study participants below the median BMI. In the lower BMI group, lean participants with high serum ALT levels who died or incurred high medical costs may have had chronic liver disease (except for fatty liver) associated with low BMI in the baseline survey. Hepatitis C virus (HCV) or hepatitis B virus infections are found more frequently in the Japanese population compared with Western populations, especially in the elderly.^22^^-^^24^ In Japan, hepatocellular carcinoma (HCC) is a major cause of death due to cancer, and HCV is the most important cause of hepatocarcinogenesis following a lengthy prodromal stage manifesting itself as chronic hepatitis and/or liver cirrhosis.^25^ Recently, Tanaka, et al^26^ suggested that an increased serum ALT level was a predictive marker for developing HCC in a 9-year follow-up study of 1,927 individuals with HCV antibody. Although they did not evaluate baseline BMI, they found that serum cholesterol levels, which usually show a positive relationship with BMI,^27^ were inversely associated with the incidence of HCC.^26^ Furthermore, malnutrition such as low BMI frequently occurs in patients with chronic liver disease such as liver cirrhosis, leading to an increase in mortality.^28^ These results might be consistent with our study findings, which identified significant increases in mortality and medical costs in participants with high serum ALT levels and low BMI.

The present study has several limitations. First, although the participants were selected from a community-based population whose health status was relatively typical of the overall Japanese population,^9^ the participants were limited to NHI beneficiaries belonging to self-employed occupational groups in one area of Shiga prefecture.^9^ The socio-economic status and lifestyle of these beneficiaries may have an effect on their health. Second, participants with a history of liver disease or current liver disease were not excluded at baseline. However, the influence of such participants on the analyses was probably relatively small, because the prevalence of clinically high levels of serum ALT was much lower than that normally observed in the Japanese population. Third, details regarding cause of death, medical diagnoses such as HCV infection, and medical treatment status were not available for the present study. As a result, we were unable to clearly identify what disease(s) directly led to increases in mortality and medical costs in participants with high serum ALT levels. Finally, we could not clearly identify a reason why the hazard ratio in the “20≤ALT<30” category was significantly lower than in the “ALT<20” category in the lower BMI group. This event may have occurred by chance.

In conclusion, in the Japanese population, it may be more useful for prognostic predictions to evaluate combinations of serum ALT levels and BMI rather than evaluating serum ALT alone. Combinations of high serum ALT levels and below median BMI appear to be associated with excess mortality and medical costs, indicating that such patients have a worse prognosis than patients with median or above-median BMI.

## APPENDIX

The Health Promotion Research Committee of the Shiga NHI Organizations

Chairman: Hirotsugu Ueshima (Department of Health Science, Shiga University of Medical Science).

Participating Researchers: Shigeo Yamashita (Kohoku General Hospital), Tomonori Okamura (Department of Health Science, Shiga University of Medical Science), Yoshinori Tominaga (Kohka Public Hospital), Kazuaki Katsuyama (Otsu Public Health Center), Fumihiko Kakuno (Nagahama Public Health Center), Machiko Kitanishi (Higashiomi City Yokaichi Public Health Center).

Associate Researchers: Koshi Nakamura (Department of Health Science, Shiga University of Medical Science), Hideyuki Kanda (Department of Hygiene and Preventive Medicine, Fukushima Medical University).

Secretary Members: Yukio Tobita, Kanehiro Okamura, Kiminobu Hatta, Takao Okada, Michiko Hatanaka (the Shiga NHI Organizations).

## References

[1] Pratt DS, Kaplan MM Evaluation of abnormal liver-enzyme results in asymptomatic patients. N Engl J Med 2000; 342: 1266-71. 10781624 10.1056/NEJM200004273421707

[2] Arndt V, Brenner H, Rothenbacher D, Zschenderlein B, Fraisse E, Fliedner TM Elevated liver enzyme activity in construction workers: prevalence and impact on early retirement and all-cause mortality. Int Arch Occup Environ Health 1998; 71: 405-12. 9766914 10.1007/s004200050299

[3] Kim HC, Nam CM, Jee SH, Han KH, Oh DK, Suh I Normal serum aminotransferase concentration and risk of mortality from liver diseases: prospective cohort study. BMJ 2004; 328: 983 (Epub 2004 Mar 17) 15028636 10.1136/bmj.38050.593634.63 PMC404493

[4] Saito Y, Yagyu K, Hattori Y, Ohno K, Okamoto N, Takahashi A, A study on fatty liver in health examination participants. Nippon Eiseigaku Zasshi 1989; 44: 953-61. (in Japanese) 2642191 10.1265/jjh.44.953

[5] Dam-Larsen S, Franzmann M, Andersen IB, Christoffersen P, Jensen LB, Sorensen TI, Long term prognosis of fatty liver: risk of chronic liver disease and death. Gut 2004; 53: 750-5. 15082596 10.1136/gut.2003.019984 PMC1774026

[6] Chitturi S, Abeygunasekera S, Farrell GC, Holmes-Walker J, Hui JM, Fung C, NASH and insulin resistance: Insulin hypersecretion and specific association with the insulin resistance syndrome. Hepatology 2002; 35: 373-9. 11826411 10.1053/jhep.2002.30692

[7] Angelico F, Del Ben M, Conti R, Francioso S, Feole K, Maccioni D, Non-alcoholic fatty liver syndrome: a hepatic consequence of common metabolic diseases. J Gastroenterol Hepatol 2003; 18: 588-94. 12702052 10.1046/j.1440-1746.2003.02958.x

[8] Ford ES The metabolic syndrome and mortality from cardiovascular disease and all-causes: findings from the National Health and Nutrition Examination Survey II Mortality Study. Atherosclerosis 2004; 173: 309-14. 15064107 10.1016/j.atherosclerosis.2003.12.022

[9] Health and Welfare Statistics Association. 2001 Kokumin Eisei no Doko (Trend for National Health and Hygiene, Japan), Tokyo: Health and Welfare Statistics Association, 2001. (in Japanese)

[10] Shiga Prefectural Government. Heisei 2-nendo Shiga-ken Tokeisyo (1990 Data of Shiga Prefecture), Otsu: Shiga Prefectural Government, 1992. (in Japanese)

[11] The Ministry of Health and Welfare. Manual for Health Check-ups under Medical Service Law for the Aged. Tokyo: Japan Public Health Association, 1987. (in Japanese)

[12] Carr DB, Utzschneider KM, Hull RL, Kodama K, Retzlaff BM, Brunzell JD, Intra-abdominal fat is a major determinant of the National Cholesterol Education Program Adult Treatment Panel III criteria for the metabolic syndrome. Diabetes 2004; 53: 2087-94. 15277390 10.2337/diabetes.53.8.2087

[13] Shimamoto K Epidemiologic study on metabolic syndrome--comparison between Japan and western countries. Nippon Rinsho 2004; 62: 1053-8. (in Japanese) 15206141

[14] Zhou BF, Stamler J, Dennis B, Moag-Stahlberg A, Okuda N, Robertson C, ; INTERMAP Research Group Nutrient intakes of middle-aged men and women in China, Japan, United Kingdom, and United States in the late 1990s: the INTERMAP study. J Hum Hypertens 2003; 17: 623-30. 13679952 10.1038/sj.jhh.1001605 PMC6561109

[15] Tanaka H, Iso H, Yokoyama T, Yoshiike N, Kokubo Y. Major health problems (cerebrovascular disease). In: Detels R, McEwen J, Beaglehole R, Tanaka H, eds. Oxford Textbook of Public Health 4th ed, Vol 3. Oxford: Oxford University Press, 2001: 1193-226.

[16] Tanaka H, Date C, Chen H, Nakayama T, Yokoyama T, Yoshiike N, A brief review of epidemiological studies on ischemic heart disease in Japan. J Epidemiol 1996; 6: S49-59. 8800274 10.2188/jea.6.3sup_49

[17] Ueshima H, Tatara K, Asakura S Declining mortality from ischemic heart disease and changes in coronary risk factors in Japan, 1956-1980. Am J Epidemiol 1987; 125: 62-72. 3788955 10.1093/oxfordjournals.aje.a114512

[18] Ueshima H Changes in dietary habits, cardiovascular risk factors and mortality in Japan. Acta Cardiol 1990; 45: 311-27. 2239030

[19] Okamura T, Kadowaki T, Hayakawa T, Kita Y, Okayama A, Ueshima H; Nippon Data80 Research Group What cause of mortality can we predict by cholesterol screening in the Japanese general population? J Intern Med 2003; 253: 169-80. 12542557 10.1046/j.1365-2796.2003.01080.x

[20] Okamura T, Hayakawa T, Kadowaki T, Kita Y, Okayama A, Elliott P, ; NIPPONDATA80 Research Group Resting heart rate and cause-specific death in a 16.5-year cohort study of the Japanese general population. Am Heart J 2004; 147: 1024-32. 15199351 10.1016/j.ahj.2003.12.020

[21] Kita Y, Okayama A, Ueshima H, Wada M, Nozaki A, Choudhury SR, Stroke incidence and case fatality in Shiga, Japan 1989-1993. Int J Epidemiol 1999; 28: 1059-65. 10661648 10.1093/ije/28.6.1059

[22] Tanaka H, Tsukuma H. Hepatitis C virus. In: Tooze J, eds. Cancer Surveys, Vol33. Infections and Human Cancer. New York: Cold Springer Laboratory Press, 1999: 213-35.

[23] Chen CJ, Wang LY, Yu MW Epidemiology of hepatitis B virus infection in the Asia-Pacific region. J Gastroenterol Hepatol 2000; 15: S3-6. 10.1046/j.1440-1746.2000.02124.x 10921373

[24] Hayashi J, Hirata M, Nakashima K, Noguchi A, Kashiwagi S, Matsui M, Hepatitis C virus is a more likely cause of chronic liver disease in the Japanese population than hepatitis B virus. Fukuoka Igaku Zasshi 1991; 82: 648-54. 1783356

[25] Kiyosawa K, Umemura T, Ichijo T, Matsumoto A, Yoshizawa K, Gad A, Hepatocellular carcinoma: Recent trends in Japan. Gastroenterology 2004;127: S17-26. 15508082 10.1053/j.gastro.2004.09.012

[26] Tanaka H, Tsukuma H, Yamano H, Oshima A, Shibata H Prospective study on the risk of hepatocellular carcinoma among hepatitis C virus-positive blood donors focusing on demographic factors, alanine aminotransferase level at donation and interaction with hepatitis B virus. Int J Cancer 2004; 112: 1075-80. 15386355 10.1002/ijc.20507

[27] Mokdad AH, Ford ES, Bowman BA, Dietz WH, Vinicor F, Bales VS, Prevalence of obesity, diabetes, and obesity-related health risk factors, 2001. JAMA 2003; 289: 76-9. 12503980 10.1001/jama.289.1.76

[28] Alberino F, Gatta A, Amodio P, Merkel C, Di Pascoli L, Boffo G, Nutrition and survival in patients with liver cirrhosis. Nutrition 2001; 17: 445-50. 11399401 10.1016/s0899-9007(01)00521-4

